# The complete mitochondrial genome of *Salix polaris,* a specie in harsh arctic environment

**DOI:** 10.1080/23802359.2020.1823269

**Published:** 2020-09-29

**Authors:** Li Zhang, Xiong Chen, Yuan Huang

**Affiliations:** School of Life Sciences, Yunnan Normal University, Kunming, Yunnan, P. R. China

**Keywords:** Mitochondrial genome, *Salix polaris*, Artic region, phylogenomic analysis

## Abstract

*Salix polaris* is a dwarf species of the genus Salix which distribute in harsh arctic environment. In this study, we sequenced and assembled whole mitochondrial genome of *S. polaris* for the first time. The complete mitochondrial genome sequence of this species is a circular molecule of 562920 bp in size, encoding 23 CDS, 21 tRNA genes, and 3 rRNA genes. Mitogenome based phylogenetic tree showed that *Salix polaris* clustered in the robust clade consist of all *Salix* species sampled in this study. The complete mitochondrial genome of *Salix polaris* adds new data into the whole mitochondrial genome of *Salix*, which is still scarce so far, and we anticipate the accumulation of mitogenome of *Salix* will facilitate phylogenetic study for this systematic difficult genus.

*Salix polaris* Wahlenb. is a member of the genus *Salix* with unique morphological traits, i.e. its a procumbent, dwarf shrub willow plant with typical heights usually less than 9 cm, which distribute in harsh environment of arctic region (Argus 1997). This species is an important element of the arctic tundra flora (Skvortsov 1999).

In this study, we sequenced and assembled the complete mitochondrial genome of *S. polaris*. The leaf sample was collected from Svalbard, Norway and dried with silica gel. The voucher specimen was deposit in the Herbarium of Kunming Institute of Botany (accession number: S.G. Wu & al. s.n.) (Chen et al. 2010). We used a modified CTAB method (Porebski 1997) to isolate the target genomic DNA. The genomic DNA was fragmented and used to construct a short-insert library following the manufacturer’s protocol (Illumina Inc., USA), and then paired-end sequenced on an Illumina Hiseq X Ten sequencer platform. A total of ca. 31.6 million reads generated and assembled by NOVOPlasty v2.7.2 with a *k*-mer of 39 (Dierckxsens et al. 2016). The raw data has been uploaded to NCBI Sequence Read Archive (SRA), the SRA number is SRR:12534577. The complete mitochondrial genome was annotated by Geneious v2020.1.1 software (Kearse et al. 2012) using the *Salix purpurea* as reference.

The mitochondrial genome of *S. polaris* was assembled as a closed-circular molecule of 5,62,920 b in length (GenBank accession No.: MT806746), containing 23 protein-coding genes (CDS), 21 tRNA genes, and 3 rRNA genes. The overall base composition is 27.4% A, 27.6% T, 22.4% C, 22.7% G, and AT content is higher than GC content. The AT content of protein-coding genes, tRNA genes, rRNA genes was 57.4, 60.2, 60.1%, respectively. All five categories of mitochondrial protein-coding genes that are typically conserved among plants (Chen et al. 2017) are present in our assembled genome.

To perform phylogenetic inference based on mitogenome, we download published Salicaceae mitochondrial whole genome (Chen et al. 2019; Choi et al. 2017; Huang et al. 2019; Kersten et al. 2016; Wei et al. 2016; Ye et al. 2017). Maximum-likelihood phylogenetic tree was conducted using IQ_TREE v1.6.10 with 10000 replicates (Nguyen et al. 2015) based on 22 mitochondrial coding sequence (CDS) shared by all seven Salicaceae species used in this study which were aligned by software MAFFT v7.47 (Katoh and Standley 2013). The best-fitted model is F81 + F + I in terms of Bayesian information criterion using Model Finder (Kalyaanamoorthy et al. 2017). We compared the phylogenetic trees of seven species complete mitochondrial genomes of Salicaceae (Chen et al. 2019; Huang et al. 2019). Our result based on mitogenome phylogenomics showed that all five *Salix* species and two outgroup *Populus* species each formed robust monophyletic clade which is sister to each other, confirmed the membership of *Salix polaris* of the *Salix* genus and consistent with former phylogeny study of Salicaceae (Chen et al, 2010; Huang et al. 2017). *Salix* is a taxa difficult in systematics, we believe that the complete mitochondrial genome of *Salix polaris* adds new data into the whole mitochondrial genome of *Salix*, which is still scarce so far, and we anticipate the accumulation of mitogenome of *Salix* species will facilitate phylogenetic study for this systematic difficult genus.

## Data Availability

The data of our research results are available in GenBank at https://www.ncbi.nlm.nih.gov/, Accession number MT806746, SRR12534677. ML phylogenetic tree of *S. Polaris* and six Salicaceae species based on CDS shared by all these seven mitochondrial complete genome, branch supports values were reported as SH-aLRT/UFboot.
